# Karyotype and cytogenetic mapping of 9 classes of repetitive DNAs in the genome of the naked catfish *Mystus bocourti* (Siluriformes, Bagridae)

**DOI:** 10.1186/1755-8166-6-51

**Published:** 2013-11-22

**Authors:** Weerayuth Supiwong, Thomas Liehr, Marcelo B Cioffi, Arunrat Chaveerach, Nadezda Kosyakova, Krit Pinthong, Tawatchai Tanee, Alongklod Tanomtong

**Affiliations:** 1 Jena University Hospital, Friedrich Schiller University, Institute of Human Genetics, Kollegiengasse 10, Jena D-07743, Germany; 2 Departamento de Genética e Evolução, Universidade Federal de São Carlos, São Carlos, SP Brazil; 3 Department of Biology Faculty of Science, Khon Kaen University, 123 Mitraphap Highway, Khon Kaen 40002, Muangkhonkaen District, Thailand; 4 Faculty of Science and Technology, Surindra Rajabhat University, 186 Moo 1, Surin 32000, Maung District, Thailand; 5 Faculty of Environment and Resource Studies, Mahasarakham University, Muang 44000, Mahasarakham, Thailand

**Keywords:** Fish cytogenetics, Major + minor rDNA sites heterochromatin, Molecular cytogenetics

## Abstract

**Background:**

In the present study, conventional and molecular cytogenetic studies were performed in the naked catfish *Mystus bocourti* (Siluriformes, Bagridae). Besides the conventional Giemsa staining, fluorescence *in situ* hybridization (FISH) using nine classes of repetitive DNAs namely 5S and 18S rDNAs, U2 snRNA, the microsatellites (CA)_15_ and (GA)_15_, telomeric repeats, and the retrotransposable elements Rex1, 3 and 6. was also performed.

**Results:**

*M. bocourti* had 2n = 56 chromosomes with a karyotype composed by 11 m + 11 sm + 6 st/a and a fundamental number (NF) equal to 100 in both sexes. Heteromorphic sex chromosome cannot be identified. The U2 snRNA, 5S and 18S rDNA were present in only one pair of chromosomes but none of them in a syntenic position. Microsatellites (CA)_15_ and (GA)_15_ showed hybridization signals at subtelomeric regions of all chromosomes with a stronger accumulation into one specific chromosomal pair. FISH with the telomeric probe revealed hybridization signals on each telomere of all chromosomes and interstitial telomeric sites (ITS) were not detected. The retrotransposable elements Rex1, 3 and 6 were generally spread throughout the genome.

**Conclusions:**

In general, the repetitive sequences were not randomly distributed in the genome, suggesting a pattern of compartmentalization on the heterochromatic region of the chromosomes. Little is known about the structure and organization of bagrid genomes and the knowledge of the chromosomal distribution of repetitive DNA sequences in *M. bocourti* represents the first step for achieving an integrated view of their genomes.

## Background

**Figure 1:**
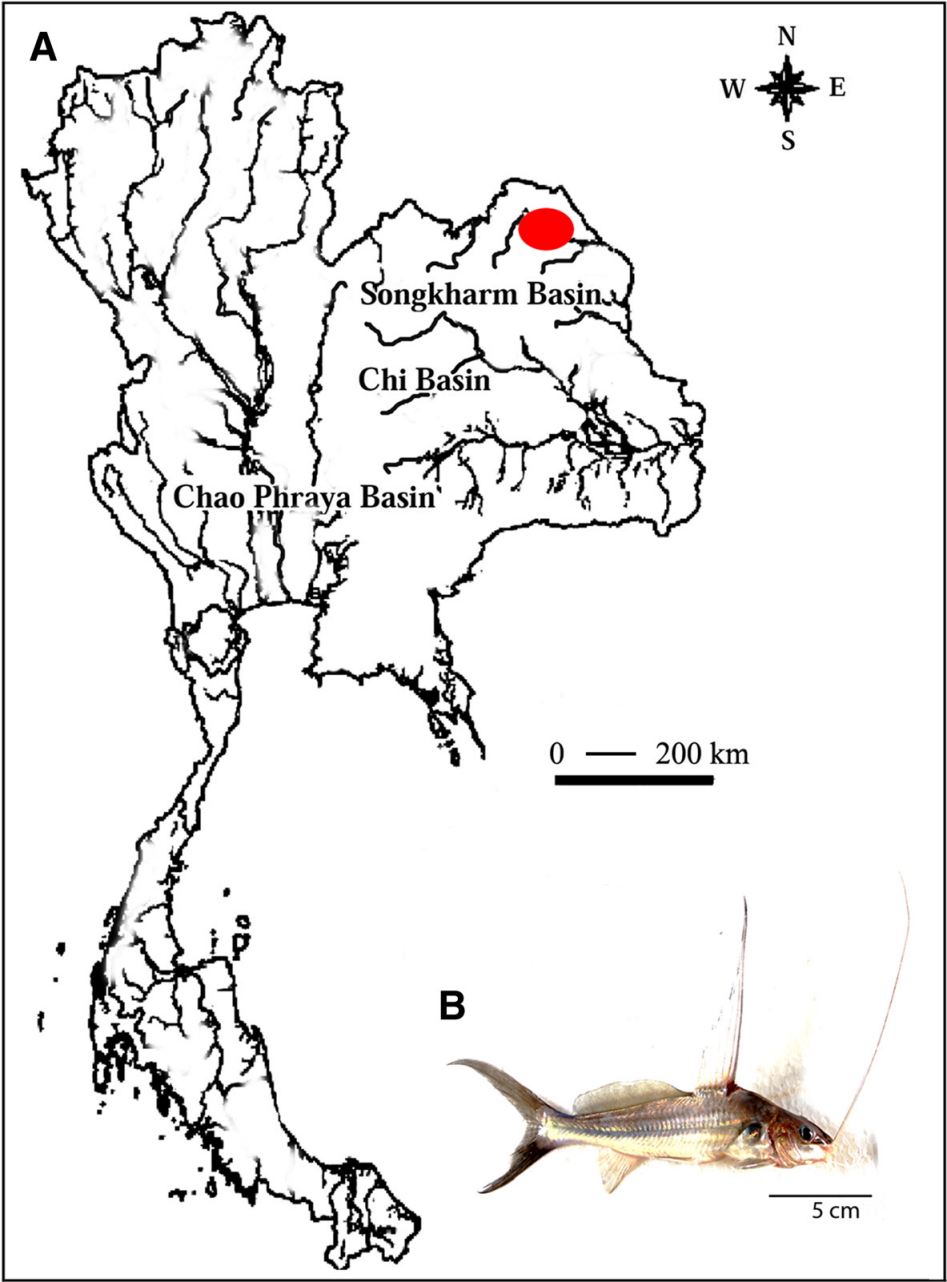
**Collection sites of Mystus bocourti. (A)** Map of Thailand indicating the collection sites and **(B)** an individual of *Mystus bocourti.* Bar = 5 cm.

Fishes of the Bagridae family belong to the order Siluriformes and are highly valued on the international fish market and represent promising species for aquaculture [1]. They are distributed in both Africa and Asia, from Japan to Borneo, including Thailand. The species *Mystus bocourti,* commonly named as hi fin *Mystus,* is a medium-size bagrid, endemic to Chao Phraya and Mekong River basins [2]. It can be distinguished from all other *Mystus* by its extraordinary high dorsal fin, involving great elongation of the non-serrate dorsal fin spine and first three or four soft rays [2] (Figure 1B). This species has been considered as endangered due to a predicated population decrease (more than 30%) in the past ten years [3]. The high levels of pollution and hydrological alterations, including dams, are some responsible for such population decrease in both the Chao Phraya and Mekong River basins [3]. There is one report based on convetionally Giemsa-stained chromosomes of *M. bocourti* showing 2n = 56 [4], and molecular cytogenetics techniques have never been applied on this species.

Remarkably, a substantial fraction of any eukaryotic genome consists of repetitive DNA sequences including multigene families, satellites, microsatellites and transposable elements [5]. Recently, the molecular cytogenetic studies using fluorescence *in situ* hybridization (FISH) for mapping repetitive DNA sequences have provided important contributions to the characterization of the biodiversity and the evolution of several fish groups [6].

This report characterizes the karyotype and the *in situ* localization of nine classes of repetitive DNA sequences (including 5S and 18S rDNAs, U2 snRNA, the microsatellites (CA)_15_ and (GA)_15_, telomeric repeats, and the retrotransposable elements Rex1, 3 and 6) on the chromosomes of *M. bocourti*.

## Results

### Karyotype

**Figure 2:**
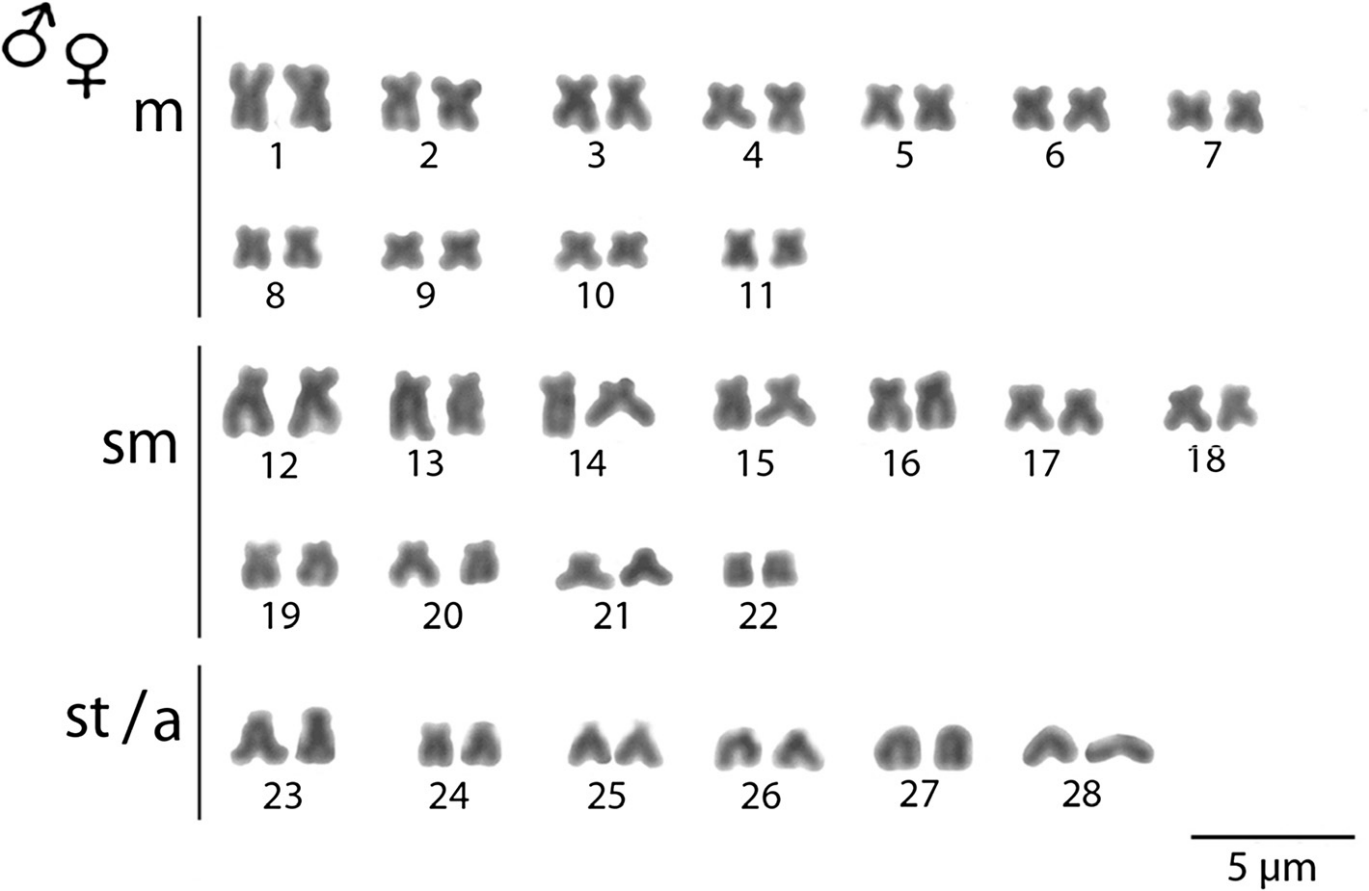
**Karyotype of *Mystus bocourti *arranged from Giemsa stained chromosomes.** Bar = 5 μm.

The *M. bocourti* individuals under study showed 2n = 56 chromosomes with the karyotype composed of (11 m + 11 sm + 6 st/a) and the fundamental number (NF) equal to 100 in both sexes, without morphologically differentiated sex chromosomes (Figure 2).

### Physical mapping of repetitive sequences

**Figure 3:**
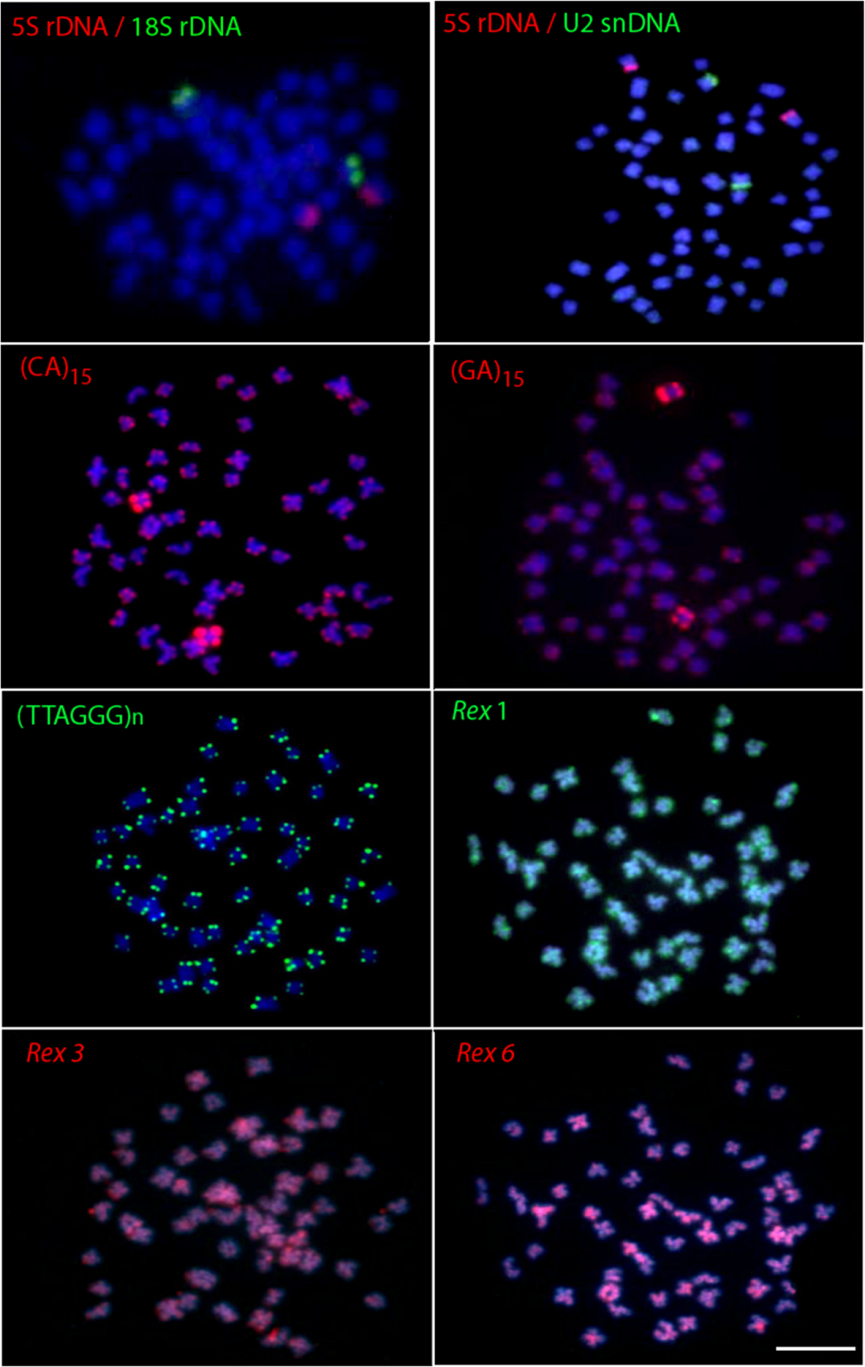
**Fluorescence *in situ *hybridization with various repetitive DNA probes on metaphase chromosomes of the naked catfish *Mystus bocourti*.** Bar = 5 μm.

Simultaneous detection of 18S and 5S rRNA by dual-colour FISH showed that both genes are located in the telomeric position of two distinct sm chromosomal pairs, not occupying a syntenic position. The same pattern was found for the U2 snRNA gene, which was located in the short arm of one sm chromosomal pair (Figure 3).

Microsatellites (CA)_15_ and (GA)_15_ showed hybridization signals at subtelomeric regions of all chromosomes with a stronger accumulation on one chromosomal pair. FISH with the telomeric probe (TTAGGG)_n_ revealed hybridization signals on each telomere of all chromosomes and interstitial telomeric sites (ITS) were not found (Figure 3). FISH using PCR fragments of retrotransposons Rex1, Rex3 and Rex6, presented a similar dispersion pattern in which the signals were broadly distributed over the whole genome including euchromatic and heterochromatic regions. However, stronger signals could be observed at telomeric region of some chromosomes (Figure 3).

## Discussion

### Karyotype

*M. bocourti* had 2n = 56 as reported in previous study [4], which is in accordance with the previous study conducted by [4]. Such 2n is also same as for the other species of the Bagridae family namely *Coreobagrus ichikawai, Hemibagrus menoda, H. nemurus* (from India), *M. albolineatus, M. singaringan, Pelteobagrus nudiceps* and *Sperata acicularis*[4,7]. However, the karyotype of the species under study was composed by 22 m + 22 sm + 12 st/a chromosomes, which differs from the previous study by [4] that reported the karyotype of *M. bocourti* consisting of 24 m + 18 sm + 14 st/a chromosomes. This fact suggests that some pericentic inversions have occurred in the karyotype differentiation of this species. In fact, the occurrence of chromosomal rearrangements has been considered a relatively common evolutionary mechanism inside the Bagridae family reviewed [7].

Karyotype diversification processes in species are subject to multiple factors, whether intrinsic (genomic or chromosomal particularities) or extrinsic (historic contingencies). Among these, restricted gene flow between populations is an important factor for fixation of karyotype changes. For example, after the occurrence of an inversion, it can be lost in the polymorphic state or, under the proper conditions, spread in the population until it is fixed. Inversions maintain areas of imbalance between alleles in loci within or influenced by these rearrangements, leading to an adaptive condition, primarily along environmental gradients. This could occur, particularly in relation to possible historical expansion and adaptation to new environments for a review see [8].

### Physical chromosome mapping of repetitive sequences

The *in situ* investigation of 9 classes of repetitive DNA sequences resulted in useful characteristics for comparative genomics at the chromosomal level, providing new insights into heterochromatin composition of the species *M. bocourti*. In fact, all of the used probes generated evident signals on both euchromatic and heterochromatic chromosomal regions, although they were preferentially located in the latter.

Ribosomal RNA genes are among the most mapped sequences in fish chromosomes. Accordingly, they can be excellent genetic markers for the comparative genomic studies, evolutionary studies as well as the genetic identification of fish species [9]. In higher eukaryotes, the ribosomal RNA genes (rDNA) comprise two repetitive DNA families, the 45S and the 5S rDNA. The 45S rDNA is formed by tandemly repeated units composed by three transcribed regions, the 18S, 5.8S, and 28S rRNA generating regions separated by internal transcribed spacers (ITS 1 and ITS 2) and by non transcribed spacer (NTS) sequences. On the other hand, the 5S rDNA is formed by tandemly repeated units of a coding region for the 5S rRNA and a non transcribed spacer (NTS) [10,11]. In *M. bocourti*, both the 5S and 18S rDNA genes mapped at the telomeric position of distinct sm chromosomal pairs, not occupying a syntenic position. This feature seems to be the most common situation revealed in fishes, and such pattern is also more common in vertebrates [12]. Since the major and 5S rDNA families are transcribed by different RNA polymerases, these functional differences may require different physical locations for these genes [12].

Another multigene family is represented by the U2 snRNA, which is one of the components of the small nuclear ribonucleoprotein particles (snRNP) and responsible for mRNA splicing [13]. The U2 snRNA was also located in the short arm of a sm chromosomal pair and not syntenic with the 5S or 18S rDNAs. This result is quite similar to the one found for the fishes *Halobatrachus didactylus*[14], *Batrachoides manglae* and *Thalassophryne maculosa*[13], but it differs from *Amphichthys cryptocentrus* and *Porichthys plectrodon*, in which the U2 snRNA signals are very widely scattered through the genome [13]. In fact, it has been proposed a trend for the U2 snRNA genes to accumulate in a specific chromosome pair over the course of the evolutionary history inside the Batrachoididae family [13]. However, in order to propose any a trend for the U2 snRNA genes accumulation in the Bagridae family, their location should be studied in more members of this family.

Microsatellites, also known as simple sequence repeats, consist of very short motifs (1–6 nucleotides in length) repeated in tandem arrays. Generally, they are located in the heterochromatic regions (telomeres, centromeres and in the sex chromosomes) of fish genomes, where a significant fraction of repetitive DNA is expected to be localized. In *M. bocourti* the microsatellites (CA)_15_ and (GA)_15_ are abundantly distributed in telomeric regions of all chromosomes and such pattern is similar to another catfishes such as, in *Imparfinis schubarti*, *Steindachneridion scripta*, and *Rineloricaria latirostris*[15]; in the zebrafish *Danio rerio*[16] and in the wolf fish *Hoplias malabaricus*[17]. However, an intriguing feature exclusive for *M. bocourti* was the strong accumulation of both microsatellites at the telomeric regions of one specific chromosomal pair, indicating that these microsatellites may be used as chromosomal markers in this fish species.

Telomeric (TTAGGG)_*n*_ sequences are present in the telomeres of vertebrate chromosomes, and the study of these sequences provides insight into the chromosomal rearrangements that have occurred during karyotype evolution of distinct organisms [6,18]. FISH with the telomeric probe (TTAGGG)_n_ revealed hybridization signals on each telomere of all chromosomes and ITS were not observed, which indicates that Robertsonian fusions or chromosomal translocations might be not involved in the karyotypic evolution of *M. bocourti.*

Transposable elements (TEs) represent another important class of repetitive DNA that is widely studied in the genome of many organisms with the Rex retrotransposon class being the most studied one within fish species reviewed in [19]. These retroelements were characterized for the first time in the genome of the swordtail fish *Xiphophorus*[20]. The *in situ* investigation of some retroelements in many species indicated that they are compartmentalized in heterochromatic regions and it can be correlated with their role in the structure and organization of centromeres or with the reduced selective pressure acting on heterochromatic regions, which are poor in gene content [21]. However, in some other fish species, despite a preferential localization to the centromeric region, TEs have a widely scattered distribution over all chromosomes, with intense hybridization signals in some specific regions [21,22]. Here, the physical mapping of different Rex elements showed that they are generally dispersed throughout the genome both heterochromatic and euchromatic regions in *M. bocourti*. Rex1 and 3 are quite accumulated in the telomeric region of several chromosomes while Rex6 showed a more dispersed pattern throughout the genome, including heterochromatin and euchromatin regions. Overall, the results indicate that TEs are important structural components of the heterochromatic regions and have played an important role in the evolutionary history of *M. bocourti* genome. Generally this distribution pattern is non-random and seems to have some relation to specific characteristics of subregions of the host genomes [23]. Importantly, retroelements increase their copy number by retrotransposition and can be substrate for homologous recombination to form various categories of DNA rearrangements including deletions, inversions, translocations, duplications and amplifications [22]. Therefore, it would be interesting to study the chromosomal distribution of Rex1*,* 3 and 6 in more members of the Bagridae family to explain the mechanism of the evolutionary dynamics of these retrotransposable elements.

## Conclusions

In general, the repetitive sequences in *M. bocourti* were not randomly distributed in the genome, suggesting a pattern of compartmentalization on the heterochromatic region of the chromosomes. In fact, a large amount of data has been generated by chromosomal mapping of repetitive DNA sequences in several fish species, providing an important source of information for the role of such sequences in the structural and functional organization of the genomes. However, little is known about the structure and organization of Bagridae fish genomes, and the knowledge of the chromosomal distribution of DNA sequences in *M. bocourti* represents the first step for achieving an integrated view of the naked catfishes genomes.

## Methods

### Materials, DNA samples and mitotic chromosome preparation

Eleven individuals of *M. bocourti* (seven males and four females) from the Songkharm River basin (Thailand), which is a branch of Mekong River, were analyzed (Figure 1A). No ethical approval was required by our institution to conduct a study on fish. Firstly, the specimens were transferred to laboratory aquaria and were kept under standard condition for 7 days prior to the experiments, which followed ethical conducts, with anesthesia being used prior to sacrificing the animals. The specimens were deposited in the fish collection of the Cytogenetic Laboratory, Department of Biology Faculty of Science, Khon Kaen University. The genomic DNA was extracted according to standard phenol–chloroform procedures [24]. Mitotic chromosomes were obtained from cell suspensions of the anterior kidney, using the conventional air-drying method [25]. Conventional staining was done using 20% Giemsa’s solution in phosphate buffer pH 6.8 for 30 minutes.

Approximately 30 metaphase spreads were analyzed to confirm the diploid chromosome number and the karyotype structure. Images were captured by the CoolSNAP system software, Image Pro Plus, 4.1 (Media Cybernetics, Silver Spring, MD, USA), coupled to an Olympus BX50 microscope (Olympus Corporation, Ishikawa, Japan). The chromosomes were classified as metacentric (m), submetacentric (sm), subtelocentric (st) or acrocentric (a) according to the arm ratios [26].

### Chromosome probes and FISH experiments

Two tandemly-arrayed DNA sequences isolated from the genome of the Erythrinidae fish *Hoplias malabaricus*, were used. The first probe contained a 5S rDNA repeat copy and included 120 base pairs (bp) of the 5S rRNA transcribing gene and 200 bp of the non-transcribed spacer (NTS) [27]. The second probe corresponded to a 1,400-bp segment of the 18S rRNA gene obtained via PCR from nuclear DNA [28]. The 5S and 18S rDNA probes were cloned into plasmid vectors and propagated in DH5α *Escherichia coli* competent cells (Invitrogen, San Diego, CA, USA). The retrotransposable elements Rex1, 3 and 6 were obtained by PCR directly from the genome of *M. bocourti* according to [20]. The U2 snDNA sequence was obtained by PCR according to [13] and the primers used were deduced from the U2 coding sequence of several model organisms available in GenBank [29].

The 18S rDNA, U2 snDNA and Rex1 probes were direct labeled with Spectrum Green-dUTP while 5S rDNA, Rex3 and Rex6 probes were direct labeled with Spectrum Orange-dUTP, all of them by nick translation according to the manufacturer’s recommendations (Roche, Mannheim, Germany).

Fluorescence *in situ* hybridization (FISH) was performed under high stringency conditions on mitotic chromosome spreads [30]. The metaphase chromosome slides were incubated with RNase (40 μg/ml) for 1.5 h at 37°C. After denaturation of chromosomal DNA in 70% formamide/2× SSC at 70°C, spreads were incubated in 2× SSC for 4 min at 70°C. The hybridization mixture (2.5 ng/μl probes, 2 μg/μl salmon sperm DNA, 50% deionized formamide, 10% dextran sulphate) was dropped on the slides, and the hybridization was performed overnight at 37°C in a moist chamber containing 2× SSC. The post hybridization wash was carried out with 1× SSC for 5 min at 65°C. A final wash was performed at room temperature in 4× SSCT for 5 min. Finally, the slides were counterstained with DAPI and mounted in an antifade solution (Vectashield from Vector laboratories).

The detection of the telomeric (TTAGGG)_n_ repeats was made with the FITC-labeled PNA probe (DAKO, Telomere PNA FISH Kit/FITC, Cat. No. K5325) and performed according to manufacturer’s recommendations.

FISH experiments with the microsatellites (CA)_15_ and (GA)_15_ as probes were performed as described in [31], with slight modifications. These sequences were directly labeled with Cy3 at 5′ terminal during synthesis by Sigma (St. Louis, MO, USA). The chromosomes were counterstained with DAPI (1.2 μg/ml), mounted in antifading solution (Vector, Burlingame, CA, USA), and analyzed in an epifluorescence microscope Olympus BX50 (Olympus Corporation, Ishikawa, Japan).

## Abbreviations

2n: Diploid chromosome number; a: Acrocentric chromosome; DAPI: 4′,6-diamidino-2-phenylindole; FISH: Fluorescence *in situ* hybridization; ITS: Interstitial telomeric sites; m: Metacentric chromosome; PCR: Polymerase chain reaction; rDNA: Ribosomal DNA; rRNA: Ribosomal RNA; sm: Submetacentric chromosome; st: Subtelocentric chromosome; TEs: Transposable elements; U2 snRNA: U2 spliceosomal small nuclear RNA.

## Competing interests

The authors declare that they have no competing interests.

## Authors’ contributions

WS and MBC carried out the molecular cytogenetic analysis and drafted the manuscript. NK, KP and TT helped in analysis and drafted the manuscript. AC and TL coordinated the study, drafted and revised the manuscript. All authors read and approved the final version of the manuscript.

## References

[B1] Nelson JS Fishes of the world 2006 4 New York: John Wiley and Sons, Inc. press

[B2] Roberts TR Systematic revision of Asian bagrid catfishes of the genus *Mystus* sensu stricto, with a new species from Thailand and Cambodia Ichthyol Explor Fishes 1994 5 241 256

[B3] Jenkins A Kullander FF Tan HH Mystus bocourti IUCN Red List of Threatened Species Version 2012.2, [http://www.iucnredlist.org]

[B4] Donsakul T Chromosome study on three species of bagrid catfishes, *Mystus albolineatus*, *M. wolffii* and *Heterobagrus bocourti*, from Thailand Proceedings of the 38th Kasetsart University Annual Conference: Fisheries and Science 2000 Bangkok: Kasetsart University

[B5] Jurka J Kapitonov VV Pavlicek A Klonowski P Kohany O Walichiewicz J Repbase update, a database of eukaryotic repetitive elements Cytogenet Genome Res 2005 110 462 467 10.1159/000084979 16093699

[B6] Cioffi MB Bertollo LAC Garrido-Ramos MA Chromosomal distribution and evolution of repetitive DNAs in fish Repetitive DNA. Genome Dynamics 2009 Basel: Karger 197 221 10.1159/000337950 22759820

[B7] Arai R Fish karyotype a check list 2011 Japan: Springer press

[B8] Hoffmann AA Rieseberg LH Revisiting the impact of inversions in evolution: from population genetic markers to drivers of adaptive shifts and speciation? Annu Rev Ecol Evol Syst 2008 39 21 42 10.1146/annurev.ecolsys.39.110707.173532 20419035 PMC2858385

[B9] Fontana F Lanfredi M Congiu L Leis M Chicca M Rossi R Chromosomal mapping of 18S–28S and 5S rRNA genes by two-colour fluorescent *in situ* hybridization in six sturgeon species Genome 2003 46 473 477 10.1139/g03-007 12834065

[B10] Long EO Dawid IB Repeated genes in eukaryotes Annu Rev Plant Physiol Plant Mol Biol 1980 49 727 764 10.1146/annurev.bi.49.070180.003455 6996571

[B11] Pendás AM Móran P Freije JP Garcia-Vásquez E Chromosomal location and nucleotide sequence of two tandem repeats of the Atlantic salmon 5S rDNA Cytogenet Cell Genet 1994 67 31 36 10.1159/000133792 8187548

[B12] Martins C Wasko AP Williams CR Organization and evolution of 5S ribosomal DNA in the fish genome Focus on Genome Research 2004 Hauppauge: Nova Science Publishers 289 318

[B13] Úbeda-Manzanaro M Merlo MA Palazón JL Cross I Sarasquete C Rebordinos L Chromosomal mapping of the major and minor ribosomal genes, (GATA)n and U2 snRNA gene by double-colour FISH in species of the Batrachoididae family Genetica 2010 138 787 794 10.1007/s10709-010-9460-1 20440541

[B14] Merlo MA Cross I Palazón JL Úbeda-Manzanaro M Sarasquete C Rebordinos L Evidence for 5S rDNA Horizontal Transfer in the toadfish *Halobatrachus didactylus* (Schneider, 1801) based on the analysis of three multigene families BMC Evol Biol 2012 12 201 10.1186/1471-2148-12-201 23039906 PMC3544641

[B15] Vanzela ALL Swarça AC Dias AL Stolf R Ruas PM Ruas CF Sbalgueiro IJ Giuliano-Caetano L Differential distribution of (GA)9 + C microsatellite on chromosomes of some animal and plant species Cytologia 2002 67 9 13 10.1508/cytologia.67.9

[B16] Shimoda N Knapik EW Ziniti J Sim C Yamada E Kaplan S Jackson D De Sauvage F Jacob H Fishman MC Zebrafish genetic map with 200 microsatellite markers Genomics 1999 58 219 232 10.1006/geno.1999.5824 10373319

[B17] Cioffi MB Kejnovsky E Bertollo LAC The chromosomal distribution of microsatellite repeats in the genome of the wolf fish Hoplias malabaricus, focusing on the sex chromosomes Cytogenet Genome Res 2011 132 289 296 10.1159/000322058 21099206

[B18] Meyne J Ratliff RL Moyzis RK Conservation of the human telomere sequence (TTAGGG)n among vertebrates Proc Natl Acad Sci USA 1989 86 18 7049 7053 10.1073/pnas.86.18.7049 2780561 PMC297991

[B19] Ferreira DC Porto-Foresti F Oliveira C Foresti F Transposable elements as a potential source for understanding fish genome Mob Genet Elements 2011 1 112 117 10.4161/mge.1.2.16731 22016858 PMC3190320

[B20] Volff JN Korting C Sweeney K Schartl M The non-LTR retrotransposon Rex3 from the fish *Xiphophorus* is widespread among teleosts Mol Biol Evol 1999 16 1427 1438 10.1093/oxfordjournals.molbev.a026055 10555274

[B21] Biémont C Vieira C Junk DNA as an evolutionary force Nature 2006 443 521 524 10.1038/443521a 17024082

[B22] Ozouf-Costaz C Brandt J Körting C Pisavo E Bonillo C Coutanceau JP Volff JN Genome dynamics and chromosomal localization of the non- LTR retrotransposons Rex1 and Rex3 in Antarctic fish Antarct Sci 2004 16 51 57 10.1017/S0954102004001816

[B23] Kidwell MG Lisch DR Transposable elements and host genome evolution Trends Ecol Evol 2000 15 95 99 10.1016/S0169-5347(99)01817-0 10675923

[B24] Sambrook J Russell DW Molecular Cloning, A Laboratory Manual 2001 3 New York: Cold Spring Harbor Laboratory Press

[B25] Nanda I Schartl M Fiechtinger W Schlupp I Parzefall J Schmid M Chromosomal evidence for laboratory synthesis of triploid hybrid between the gynogenetic teleost *Poecilia formosa* and its host species J Fish Biol 1995 47 619 623

[B26] Levan A Fredga K Sandberg AA Nomenclature for centromeric position on chromosomes Hereditas 1964 52 201 220

[B27] Martins C Ferreira IA Oliveira C Foresti F Galetti PM Jr A tandemly repetitive centromeric DNA sequence of the fish *Hoplias malabaricus* (Characiformes: Erythrinidae) is derived from 5S rDNA Genetica 2006 127 133 141 10.1007/s10709-005-2674-y 16850219

[B28] Cioffi MB Martins C Centofante L Jacobina U Bertollo LAC Chromosomal variability among allopatric populations of Erythrinidae fish *Hoplias malabaricus*: Mapping of three classes of repetitive DNAs Cytogenet Genome Res 2009 125 132 141 10.1159/000227838 19729917

[B29] Cross I Merlo A Manchado M Infante C Cañavate JP Rebordinos L Cytogenetic characterization of the Solea senegalensis (Teleostei: Pleurenectiformes. Soleidae): Ag-NOR, (GATA)n, (TTAGGG)n and ribosomal genes by one-color and two-color FISH Genetica 2006 128 253 259 10.1007/s10709-005-5928-9 17028955

[B30] Pinkel D Straume T Gray J Cytogenetic analysis using quantitative, high sensitivity, fluorescence hybridization Proc Natl Acad Sci USA 1986 83 2934 2938 10.1073/pnas.83.9.2934 3458254 PMC323421

[B31] Kubat Z Hobza R Vyskot B Kejnovsky E Microsatellite accumulation in the Y chromosome of *Silene Latifolia* Genome 2008 51 350 356 10.1139/G08-024 18438438

